# Nutritional symbionts enhance structural defence against predation and fungal infection in a grain pest beetle

**DOI:** 10.1242/jeb.243593

**Published:** 2022-01-04

**Authors:** Sthandiwe Nomthandazo Kanyile, Tobias Engl, Martin Kaltenpoth

**Affiliations:** 1 Evolutionary Ecology, Institute of Organismic and Molecular Evolution, Johannes Gutenberg-University, 55128 Mainz, Germany; 2 Department of Insect Symbiosis, Max-Planck-Institute for Chemical Ecology, 07745 Jena, Germany

**Keywords:** Bacteroidetes, *Candidatus* Shikimatogenerans silvanidophilus, Cuticle, Mutualism, *Oryzaephilus surinamensis*, Sawtoothed grain beetle, Symbiosis, Structural defence

## Abstract

Many insects benefit from bacterial symbionts that provide essential nutrients and thereby extend the hosts’ adaptive potential and their ability to cope with challenging environments. However, the implications of nutritional symbioses for the hosts’ defence against natural enemies remain largely unstudied. Here, we investigated whether the cuticle-enhancing nutritional symbiosis of the saw-toothed grain beetle *Oryzaephilus surinamensis* confers protection against predation and fungal infection. We exposed age-defined symbiotic and symbiont-depleted (aposymbiotic) beetles to two antagonists that must actively penetrate the cuticle for a successful attack: wolf spiders (Lycosidae) and the fungal entomopathogen *Beauveria bassiana*. While young beetles suffered from high predation and fungal infection rates regardless of symbiont presence, symbiotic beetles were able to escape this period of vulnerability and reach high survival probabilities significantly faster than aposymbiotic beetles. To understand the mechanistic basis of these differences, we conducted a time-series analysis of cuticle development in symbiotic and aposymbiotic beetles by measuring cuticular melanisation and thickness. The results reveal that the symbionts accelerate their host's cuticle formation and thereby enable it to quickly reach a cuticle quality threshold that confers structural protection against predation and fungal infection. Considering the widespread occurrence of cuticle enhancement via symbiont-mediated tyrosine supplementation in beetles and other insects, our findings demonstrate how nutritional symbioses can have important ecological implications reaching beyond the immediate nutrient-provisioning benefits.

## Abstract (precis)

**Summary:** Nutritional bacterial symbionts of the saw-toothed grain beetle *Oryzaephilus surinamensis* enhance host cuticle formation, conferring protection against predation and fungal infection.

## INTRODUCTION

Beneficial symbiotic associations (mutualisms) are taxonomically widespread and play a pivotal role in shaping the ecology and evolution of insects. The classification of the type of mutualism between organisms and thus the context in which it is studied has traditionally been reliant on the immediate observed effect that the symbiont has on its host. In nutritional symbioses, microbes aid host metabolism by provisioning essential nutrients (e.g. amino acids or B vitamins) that enable their hosts to subsist on nutrient-deficient diets such as vertebrate blood or plant sap (Michalkova et al., 2014; Douglas et al., 2001). Alternatively, microbial symbionts may be involved in the degradation of fastidious polymers (Salem et al., 2017) or the detoxification of noxious compounds such as phytotoxins and pesticides (Itoh et al., 2018). In doing so, symbionts have enabled insects to exploit a variety of diets, and thus occupy ecological niches that would otherwise be inaccessible.

In defensive symbioses, hosts exhibit higher fitness than symbiont-free individuals in the presence of natural enemies such as pathogens (i.e. bacteria, fungi and viruses), parasites or predators (Clay, 2014; Oliver et al., 2014; Flórez et al., 2015). The most prominent mechanistic basis of defensive symbioses involves the production of bioactive secondary metabolites with toxic or deterrent functions by the microbial partner (Clay, 2014; Oliver and Perlman, 2020). For instance, in a tripartite symbiosis, leaf cutter ants harbour antibiotic-producing bacteria of the genus *Streptomyces* that inhibit the growth of pathogenic *Escovopsis* spp. on their fungal gardens (Currie et al., 1999; 2003). Similarly, *Burkholderia* symbionts provide antifungal protection to the eggs of their host, *Lagria villosa*, by producing a concoction of antibiotics (Flórez et al., 2017; 2018), and *Streptomyces* symbionts protect immature beewolf wasps from fungal infections (Kaltenpoth et al., 2005; Kroiss et al., 2010). By contrast, symbiont-mediated predator defence in insects has, as far as we know, only been directly demonstrated in *Paederus* spp. beetles, whose bacterial symbiont *Pseudomonas aeruginosa* produces the chemical compound pederin that deters wolf spiders (Kellner and Dettner, 1996). The Asian citrus psyllid, *Diaphorina citri*, was found to harbour an obligate intracellular symbiont, *Profftella armatura*, in which 15% of its highly eroded genome is devoted to genes involved in the synthesis of a pederin-like polyketide called diaphorin (Nakabachi et al., 2013). However, while diaphorin exhibits cytotoxic activity to cultured mammalian and insect cells (Yamada et al., 2019), no studies have yet shown its effects against any natural enemy of Asian citrus psyllids. Nevertheless, it remains plausible that diaphorin is involved in the chemical defence of Asian psyllids against natural predators.

Although the production of bioactive compounds by microbes is taken as a *prima facie* criterion for the categorisation of a mutualism as defensive, there are various other ways in which microbes can assist in the protection of their hosts against antagonists. In addition to upregulating the host's immune system in a ‘vaccine-like’ manner and competitively excluding pathogenic microorganisms, symbiotic microbes can, through their nutritional contributions, improve the overall health of their host, enabling it to better invest in defence against antagonists (Clay, 2014; Little and Kraaijeveld, 2004; Flórez et al., 2015). However, nutritional symbioses are not typically studied in the context of their defensive properties.

One of the first lines of defence in insects is their cuticle, which primarily consists of a cross-linked matrix of cuticular proteins and chitin and serves as a structural barrier between the insect and its external environment (Noh et al., 2016). Importantly, the hardening and tanning of the outer layer of the cuticle (exocuticle) is reliant upon two processes: (i) sclerotization, which involves the cross-linking and stabilisation of the cuticle through the incorporation of phenolic compounds, resulting in stiffness or rigidity of the cuticle; and (ii) melanisation, in which melanin is deposited within the cuticle, resulting in pigmentation (Andersen, 2010; Noh et al., 2016; Evison et al., 2017). At the centre of both processes is the hydroxylation of the aromatic amino acid tyrosine into 3,4-dihydroxyphenylalanine (DOPA). In the case of beetles, which have a particularly hardened cuticle and strongly sclerotised front wings (elytra), a substantial investment in cuticle biosynthesis can be expected (Noh et al., 2016). However, insects are unable to synthesise the benzene ring of aromatic amino acids (Evison et al., 2017). Hence, they must obtain these compounds via their diet or by partnering with microbes that can produce aromatics via the shikimate pathway (Evison et al., 2017). Indeed, tyrosine-provisioning symbionts have been reported across multiple different beetle taxa: in the black hard weevil *Pachyrhynchus infernalis* (Anbutsu et al., 2017; Anbutsu and Fukatsu, 2020), in the cereal weevils *Sitophilus* spp. (Oakeson et al., 2014; Vigneron et al., 2014), in the grain pest beetle *Oryzaephilus surinamensis* (Engl et al., 2018; Hirota et al., 2017; Kiefer et al., 2021), and in the West Indian sweet potato weevil, *Euscepes postfasciatus* (Kuriwada et al., 2010). Similar observations of symbiont-assisted cuticle biosynthesis have additionally been reported in carpenter ants *Camponotus fellah* (Sinotte et al., 2018), in the invasive ant species *Cardiocondyla obscurior* (Klein et al., 2016) and in turtle ants *Cephalotes varians* (Duplais et al., 2021, Jackson et al., 2021). In several of these cases, experimental depletion of the symbionts was shown to result in a phenotype with reduced cuticle thickness and/or changes in cuticular pigmentation.

*Oryzaephilus surinamensis* is a cosmopolitan pest of stored grain (Howard et al., 1995) that harbours the intracellular Bacteroidetes symbiont *Candidatus* Shikimatogenerans silvanidophilus (henceforth *Shikimatogenerans*), which supplements the host with the tyrosine precursor prephenate, thereby assisting in cuticle formation (Hirota et al., 2017; Engl et al., 2018; Kiefer et al., 2021). Concordantly, experimentally symbiont-depleted (aposymbiotic) beetles exhibit a thinner and less melanised cuticle than their symbiotic counterparts (Hirota et al., 2017; Engl et al., 2018) and show a reduced resistance to desiccation and lower fitness under dry ambient conditions (Engl et al., 2018). However, besides delayed reproductive maturation in symbiotic beetles, no further differences between symbiotic and aposymbiotic beetles regarding other life-history traits were observed in laboratory rearing conditions (Engl et al., 2020).

Here, we set out to investigate a possible symbiont contribution to the defence of *O. surinamensis* against two natural enemies: wolf spiders (Lycosidae), which are widely distributed generalist predators, and the entomopathogenic fungus *Beauveria bassiana*. Like other predators and many entomopathogenic fungi, these natural enemies must overcome the insects’ cuticle for successful predation or infection. We thus hypothesised that (1) symbiont elimination results in higher predation pressure and reduced handling times by spiders as a result of a thinner and less sclerotised cuticle, and (2) symbiont-deprived beetles are more susceptible to entomopathogenic fungi which infect the host through the cuticle. To test these predictions, we exposed age-defined symbiotic and aposymbiotic beetles to wolf spiders in predation assays and *B. bassiana* in fungal bioassays and recorded their survival probability. Additionally, we conducted a time-series comparison of cuticle development in symbiotic and aposymbiotic beetles. We found that symbionts reduce their host’s predation and fungal infection risk, particularly in the first few days post-eclosion, by enabling rapid cuticle formation*.* This protective effect corresponds to a faster thickening and tanning of the cuticle, indicating that the symbionts enable their host to rapidly escape from the vulnerable post-eclosion phase.

## MATERIALS AND METHODS

### Beetle cultures

*Oryzaephilus surinamensis* (Linnaeus 1758) cultures used in this study were derived from the JKI strain (2015) of the Julius-Kühn-Institute (Berlin, Germany) and maintained in 1.8 l plastic containers on a diet of oat flakes (Huber-Mühle, Hohberg, Germany). Temperature and relative humidity were kept at 28°C and 60%, respectively. Symbionts were eliminated from a subculture using tetracycline 2 years prior to the start of the experiments to obtain aposymbiotic beetles as described in Engl et al. (2018). The symbiont status of the lines is routinely checked using established DNA extraction and qPCR protocols (see Engl et al., 2018).

Age-defined adult beetles were obtained by separating aposymbiotic and symbiotic pupae into 24-well plates, lined with Fluon (Sigma-Aldrich, Hamburg, Germany) to prevent the escape of beetles. A single oat flake was placed in each well and the plates were observed daily, until the day of adult emergence was recorded.

### Cuticle development

The effect of symbiont presence or absence on the development of the cuticle during the first 7 days post-pupal eclosion was evaluated using two parameters, i.e. cuticular melanisation and cuticle thickness as described by Engl et al. (2018). Briefly, 9–12 beetles from each treatment per age group (day) were anaesthetised by being chilled on ice, photographed with an RGB colour camera (Axiocam 208, Zeiss, Jena, Germany) mounted to a StereoDiscovery V.8 dissection scope (Zeiss) and fitted with a constant intensity light source (SLIM-LED S40-75, Schott, Germany) under identical software parameters. The software Natsumushi (Tanahashi and Fukatsu, 2018) was used to measure average red values in a defined circular area of the thorax. After being photographed, 6–9 beetles from each treatment per age group were fixed in phosphate-buffered 4% formaldehyde (Carl Roth, Karlsruhe, Germany), dehydrated and then embedded with Technovit^®^ 8100 (Kulzer, Germany). Semi-thin cross-sections (8 μm) of the thorax were obtained using a microtome and mounted on silanised glass slides with ROTI^®^Histokitt (Carl Roth). To measure cuticle thickness, images were taken with an Axiocam 506 (Zeiss) under differential interference contrast at 200× magnification on an AxioImager.Z2 (Zeiss). One dorsal, ventral and lateral point, respectively, were randomly chosen to measure the diameter of the cuticle using the Zen software distance tool.

### Predation assays

Adult wolf spiders (*Pardosa* spp., Lycosidae) were collected on the campus of the Johannes Gutenberg University, Mainz, Germany. Upon arrival in the laboratory, spiders were immediately given two 5th instar *O. surinamensis* larvae to standardise hunger levels and were then kept at 20°C and 60% relative humidity. Spiders were then subjected to an initial 7 day period of starvation before the start of the experiments and were given water *ad libitum* by spraying. Round plastic containers (diameter 5 cm), with white filter paper taped on the bottom to provide traction, were used as assay arenas. After every assay, the containers were wiped with 70% ethanol and the filter paper was replaced to eliminate possible olfactory cues left from a previous assay (Linz et al., 2016). Spiders (*n*=39) were presented with adult beetles of ascending age (1–7 days old) from both treatment groups (symbiotic, *n*=62; and aposymbiotic, *n*=64). Thus, each spider encountered beetles in the order: 1 day old aposymbiotic and 1 day old symbiotic, 2 day old aposymbiotic and 2 day old symbiotic and so forth. The order of treatments remained unchanged, such that spiders were always given aposymbiotic beetles first in each age group. Even though spider age thus correlated with prey age in our assays, we chose this design to prevent possible learning effects that otherwise may have led spiders to reject beetles based on previous experience with strongly sclerotised and melanised individuals. Additionally, as some spiders did not survive the entire duration of the experiments (presumably due to old age when initially being collected from the field), new spiders were subsequently collected to complete the assays, and given beetles of ascending age from the point where a previous spider died. Between trials, each spider was starved for at least 5 days and spider motivation to attack (quantified as ‘latency’, i.e. the time taken for a starving spider to attack a beetle once the beetle was introduced into the arena after 5 days of starvation) was not affected by beetle symbiont status (Fig. S1). Spiders that rejected beetles in assays were immediately given a 5th instar larva as a hunger control. The assays were conducted as described in Linz et al. (2016). Briefly, a spider was introduced into the arena and allowed to acclimatise for 5 min. Using a plastic Falcon tube (Eppendorf, Hamburg, Germany), the spider was confined to one end of the arena and an age-defined beetle was introduced into the arena. After a further 5 min of acclimatisation, the beetle was also contained at the opposite end of the arena. The trial started with the simultaneous removal of both Falcon tubes and the interaction was observed. The following behavioural definitions applied. (i) Attack – defined as physical contact between the spider and beetle, where the spider grabs and picks up the beetle. (ii) Survived – when the beetle was attacked and then dropped by the spider. Following survival, beetles were removed from the assay arena and placed in a separate container with oats and further observed for injuries. There were only two instances where surviving beetles had sustained visible injuries. Furthermore, it was observed in pilot experiments that beetles were never attacked more than once. Spiders ignored beetles which they were unable to kill at the first attack, even if the beetle was left with the spider in the arena for extended periods of time. (iii) Rejection – defined as an event where a spider did not engage with the beetle at all (no attack event).

In the case of successful attacks, the spiders remained in the arena until they finished eating the beetle. In the case of rejection, spiders remained in the assay arena with the beetle for 1 h hour before they were removed. The following variables were recorded during the assays: (i) the time taken for the spider to attack the beetle and (ii) whether the beetle survived an attack encounter or not.

Spiders (*n*=28) were also presented with symbiotic (*n*=17) and aposymbiotic (*n*=18) 5th instar larvae, in assays that proceeded as described above. Here, spider handling time (defined as the time from the moment of larva capture to the end of feeding) was recorded. To assess possible differences in mass between symbiotic and aposymbiotic larvae that may influence spider handling times, we weighed randomly selected symbiotic (*n*=17) and aposymbiotic (*n*=19) 5th instar larvae using an electronic scale (Precisa ES 225M-DR, Dietekon, Switzerland).

### *Beauveria bassiana* cultures and fungal bioassays

A commercial oil suspension of *B. bassiana* strain ATCC7404 was obtained from Palmruessler (Munich, Germany). The suspension was mixed with sterile distilled water and cultured on potato dextrose agar (PDA) medium (Carl Roth) at 26°C under dark conditions. After 7 days, spores were harvested from the culture plate by washing with 0.05% Triton-X (Carl Roth) and filtered (11 μm pore size) to remove hyphal fragments. Spores were then resuspended in sterile phosphate-buffered saline (1× PBS: 137 nmol l^−1^ NaCl, 2.7 mmol l^−1^ KCl, 10 mmol l^−1^ Na_2_HPO_4_, 2 mmol l^−1^ KH_2_PO_4_) and were first passaged through *O. surinamensis* to obtain virulent cultures. Beetles were exposed to *B. bassiana* ATCC7404 as follows. Clean, round plastic containers were inoculated with 25 μl of the fungal spore suspension (2.4×10^7^ spores ml^−1^ as measured with a Neubauer Chamber) and allowed to dry under sterile conditions. For controls, the container was instead inoculated with 25 μl of sterile PBS. Thereafter, 15 beetles were introduced into each container and exposed to the dry fungal spores for a period of 14 days. Oats were provided to the beetles for the duration of the experiment and these were frequently replaced with fresh oats, to prevent fungal overgrowth on the food. The small plastic containers were placed in a bigger plastic container and incubated at 27°C and 80% relative humidity. Dead beetles were immediately removed from the assay, briefly washed in 12% bleach, rinsed in sterile distilled water, placed on a moist filter paper, and incubated in the same conditions as above. Spores were re-harvested from beetle carcasses by vigorously shaking dead beetles in 0.05% Triton-X to dislodge them from the cuticle. The resuspension was then plated on PDA to observe for viability and subsequently used to reinfect beetles. After the third passage of the fungus through beetles, a final spore suspension was prepared on PDA and standardised to 2.4×10^7^ spores ml^−1^, and used to infect three replicates of newly emerged beetles (<24 h old, hereafter referred to as ‘young’ beetles) and 14 day old beetles (hereafter referred to as ‘old’ beetles; *n*=15 symbiotic and aposymbiotic beetles per treatment), and mortality was recorded for a 14 day period.

### Statistical analysis

Data were analysed using the statistical software R Studio 3.6.2 (http://www.R-project.org/). To evaluate the influence of symbiont status and age on cuticle thickness and melanisation, generalised linear models (GLMs) were fitted to the data using the ‘glm’ functions from the MASS package (Venables and Ripley, 2002). Following significant effects of symbiont status and age on cuticle thickness and melanisation, Wilcoxon rank sum tests with the Benjamini–Hochberg *P*-adjustment method (Dunn, 1964) were used for pairwise comparisons to determine differences between aposymbiotic and symbiotic beetles for each age group. To assess whether symbiont status and beetle age influenced adult and larval mortality, generalised linear mixed effects ‘glmer’ models from the lme4 package (Bates et al., 2015 preprint) were used. Binomial distribution was specified in the case of adult predation assays. The response was survival outcome (adult predation model) or handling time (larval predation model); symbiont status was introduced as a fixed effect in both models, with beetle age as an additional fixed effect for the adult predation model. Spider identity was specified as a random effect for both models. Backward model reduction was conducted to select the minimum adequate model. The influence of symbiont status on larval mass was assessed with an ANOVA. Normality and variance homogeneity assumptions for the ANOVA were tested using the Shapiro–Wilk test and the *F*-test, respectively, with data being accepted as normal and homogeneous in variance when *P*>0.05. Survival of young and old beetles in the fungal bioassays was analysed with Cox mixed-effects models of the COXME package (Therneau, 2012). Here, symbiont status and age were explanatory factors and the replicate number was introduced as a random factor. Kaplan–Meier models were used to plot survival probability from the RMS package (Harrell and Frank, 2013). Plots were illustrated using ggplot2 (Wickham, 2016).

## RESULTS

### Symbiont influence on cuticle development

**Fig. 1.**
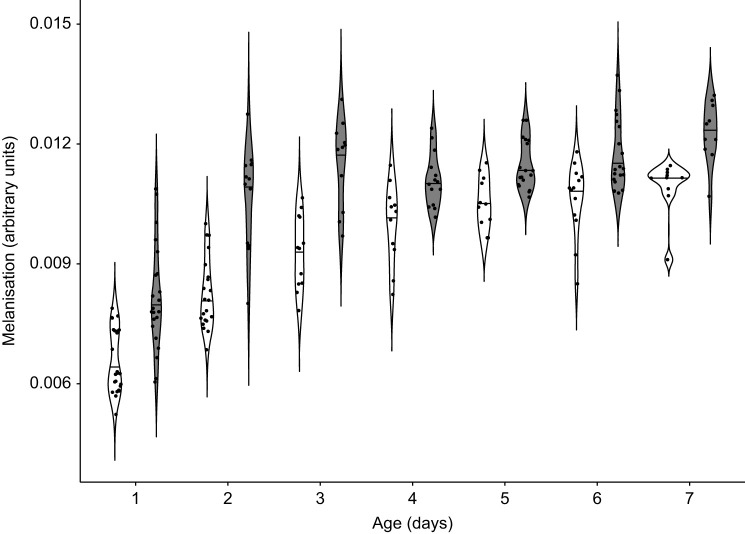
**Melanisation progression in symbiotic and aposymbiotic *Oryzaephilus surinamensis* beetles from day 1 to day** **7 post-eclosion.** Inverse red values of symbiotic (grey contours, *n*=108) and aposymbiotic (white contours, *n*=104) beetles in different age groups. Higher inverse red values reflect darker cuticular coloration. The horizontal line inside each contour represents the median. Significant differences (*P*<0.05) were observed between treatments in every age group, following Wilcoxon pairwise comparisons with the Benjamini–Hochberg *P*-adjustment method.

To gain insight into the contribution of symbionts to cuticle formation, we monitored symbiotic (*n*=108) and aposymbiotic beetles (*n*=104) during the first 7 days post-eclosion. As expected, melanisation was significantly influenced by symbiont status (Table 1; GLM, *P*<0.001) and age (Table 1; GLM, *P*<0.001). A pairwise within-treatment comparison of melanisation, across different age groups using the Wilcoxon rank sum test revealed that individuals of both treatments progressively melanised over the first 7 days, but a significant single-day increase in melanisation was only observed from day 1 to day 2 for both symbiotic and aposymbiotic beetles (comparison of day 1 with day 2: Benjamini–Hochberg corrected *P*<0.001, both treatments; Fig. 1; Fig. S2). Between-treatment comparisons revealed significant differences in melanization between symbiotic and aposymbiotic beetles for all days (Benjamini-Hochberg corrected *P*<0.05). Interestingly, by day 3, symbiotic beetles had already attained the same level of melanisation as 7 day old aposymbiotic beetles (day 3 and day 7: *W*=79, Benjamini–Hochberg corrected *P*=0.09862).

**Table 1.**
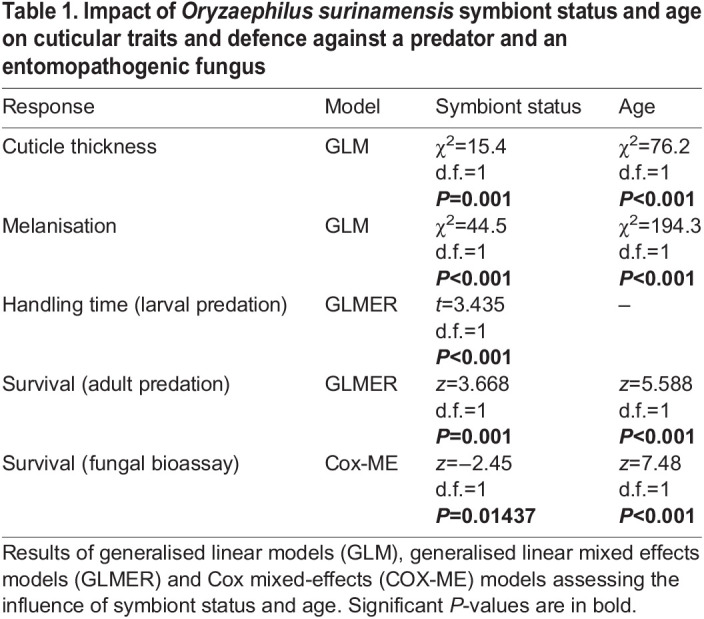
Impact of *Oryzaephilus surinamensis* symbiont status and age on cuticular traits and defence against a predator and an entomopathogenic fungus

**Fig. 2.**
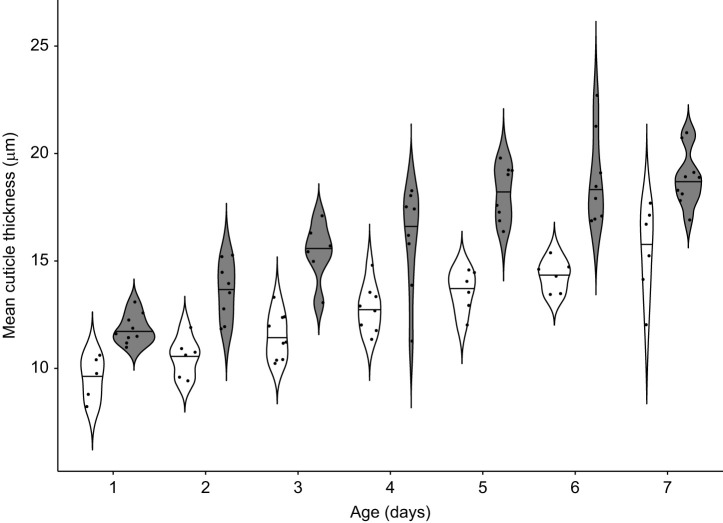
**Cuticle thickness progression in symbiotic and aposymbiotic beetles from day 1 to day 7 post-eclosion.** Mean cuticle thickness of symbiotic (grey contours, *n*=56) and aposymbiotic (white contours, *n*=46) beetles in different age groups. The horizontal line inside each contour represents the median. Significant differences (*P*<0.05) between treatments were observed in every age group, following Wilcoxon pairwise comparisons with the Benjamini–Hochberg *P*-adjustment method.

Similar results were observed for the progression of cuticle thickness (symbiotic *n*=56, aposymbiotic *n*=46). Symbiont status and age significantly influenced cuticle thickness (Table 1; *P*<0.001 for both). Within-treatment comparisons revealed that symbiotic beetles significantly increased the thickness of their cuticle within the first 3 days (Wilcoxon rank sum comparison of day 1 and day 2: Benjamini–Hochberg corrected *P*=0.0131; day 2 and day 3: Benjamini–Hochberg corrected *P*=0.0447; Fig. 2). Subsequently, cuticle thickness continued to increase until day 7, albeit at a slower and statistically insignificant rate. By contrast, the rapid day-by-day increase in cuticle thickness observed in symbiotic beetles in the first 3 days was not observed in aposymbiotic beetles (comparison of day 1 and day 2: Benjamini–Hochberg corrected *P*=0.1551; day 2 and day 3: Benjamini–Hochberg corrected *P*=0.1688; Fig. 2). Nevertheless, aposymbiotic beetles also progressively increased the thickness of their cuticle until day 7. Between-treatment comparisons revealed that symbiotic and aposymbiotic beetles never had the same cuticle thickness at any time point (Benjamini-Hochberg corrected *P*<0.05 for all days). Interestingly, by day 3, the cuticle thickness of symbiotic beetles did not significantly differ from that of 7 day old aposymbiotic beetles (*W*=20, Benjamini–Hochberg corrected *P*=0.8182).

### Symbiont influence on defence against wolf spiders

**Fig. 3.**
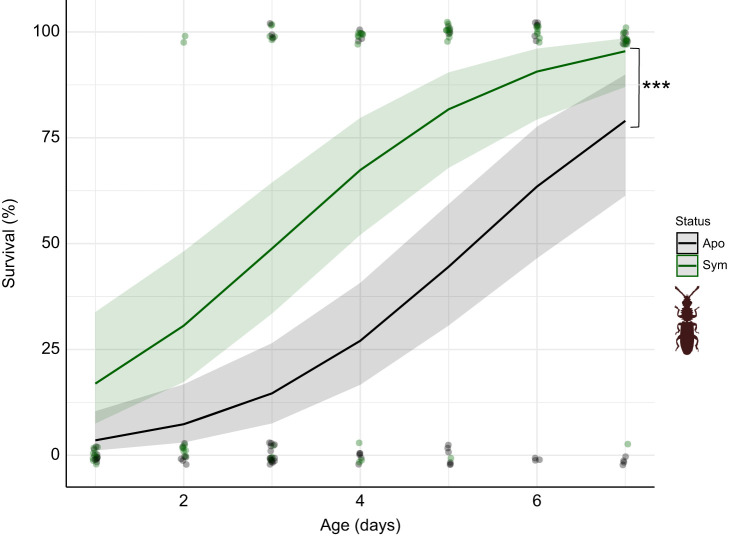
**Impact of symbiont status and age on adult beetle defence against predatory wolf spiders.** Survival probability (mean and 95% confidence interval) of symbiotic (green line and shaded area; green dots show single data points) and aposymbiotic (black line and shaded area; black dots show single data points) adult beetles of different ages as predicted by the generalised linear mixed effects model (GLMER). Both symbiont status (***P<0.001) and age (*P*<0.001) had a significant influence on survival probability (GLMER, spiders *n*=39, symbiotic *n*=62, aposymbiotic *n*=64).

We exposed age-defined symbiotic and aposymbiotic beetles to starved wolf spiders (*n*=39) in predation assays. We found that both symbiont status and age had a significant effect on beetle survival (Table 1; GLMER, *P*<0.001 for both; aposymbiotic *n*=64, symbiotic *n*=62). The interaction effect of age and symbiont status was not significant and was thus removed from the model. The age effect seemed to be particularly strong, as young beetles of both treatments had a low survival probability, but this increased with age for both treatment groups (Fig. 3). Nevertheless, the odds of survival increased significantly more rapidly for symbiotic beetles, while a more gradual trend was observed for aposymbiotic beetles. Specifically, reaching a 50% survival probability took 3 days for symbiotic beetles, but 5 days for aposymbiotic beetles (Fig. 3). Overall, symbiotic beetles suffered lower mortality than aposymbiotic beetles.

**Fig. 4.**
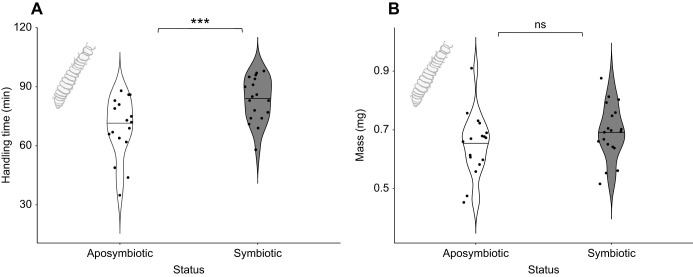
**Impact of symbiont status on handling time of larvae by predatory wolf spiders, and on larval mass.** (A) Spiders (*n*=28) took significantly longer to handle symbiotic 5th instar larvae (grey contours, *n*=18) than they did with aposymbiotic larvae (white contours, *n*=17) (GLM, ***P<0.001). (B) Differences in mass of symbiotic (grey contours, *n*=19) and aposymbiotic (white contours, *n*=17) larvae were not significant (ANOVA, ns, *P*>0.05). The horizontal line inside contours indicates the median.

A previous study (Engl et al., 2018) found a 20% reduction in cuticle thickness in aposymbiotic *O. surinamensis* larvae (4th instar). Thus, we also presented larvae (5th instar, aposymbiotic *n*=17, symbiotic *n*=18) to wolf spiders (*n*=28) in a separate experiment and measured spider handling times. Spiders always consumed the presented larva, but took significantly longer to capture and consume symbiotic beetle larvae, compared with aposymbiotic larvae (Table 1; GLM, *P*<0.001). On average, it took spiders 14 more minutes to handle symbiotic than aposymbiotic larvae (83 min and 69 min to handle symbiotic and aposymbiotic larvae, respectively; Fig. 4A). We then asked whether these differences in handling time could be due to differences in size (mass) between symbiotic and aposymbiotic larvae. We measured larval mass and found that there was a trend towards symbiotic larvae being heavier than aposymbiotic beetles (Fig. 4B), but this difference was not significant (ANOVA; aposymbiotic *n*=17, symbiotic *n*=19, d.f.=1; *F*=1.659; *P*=0.206).

### Symbiont influence on defence against *B. bassiana*

**Fig. 5.**
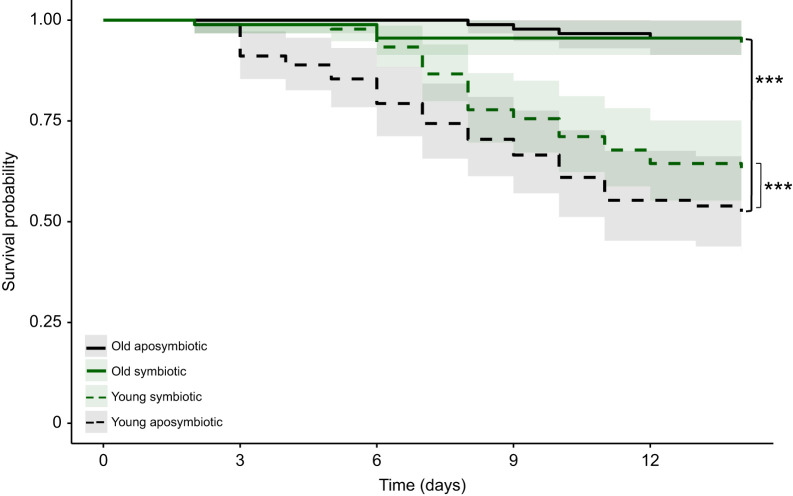
**Survival probability of young (<24 h post-eclosion) and old (14 days post-eclosion) symbiotic and aposymbiotic beetles exposed to *Beauveria bassiana* spores.** Mortality was significantly influenced by symbiont status and age (Cox mixed-effects model, *P*=0.01437 and *P*<0.001, respectively). Lines depict the mean and the shaded area the 90% confidence interval. Young beetles (dashed lines) of both treatment groups suffered significantly higher mortality than old beetles (solid lines; old aposymbiotic *n*=45, old symbiotic *n*=45; ***P<0.001), and young aposymbiotic beetles (dashed black line; *n*=45) suffered from an earlier onset of mortality as well as a higher mortality rate than young symbiotic beetles (dashed green line; *n*=45, ***P<0.001).

Symbiont influence on the defence of *O. surinamensis* against *B. bassiana* was evaluated using Cox mixed-effects models. Mortality was significantly influenced by both symbiont status and age in the group exposed to the entomopathogen (Table 1; Cox-me; *P*=0.014 and *P*<0.001, respectively; Fig. 5). The survival probability of young beetles (young aposymbiotic *n*=45, young symbiotic *n*=45) was significantly lower than that of old beetles (old aposymbiotic *n*=45, old symbiotic *n*=45) regardless of symbiont status (Table S1; Fig. 5). While mortality of old beetles did not differ between symbiotic and aposymbiotic beetles, young aposymbiotic beetles showed an earlier onset of mortality and suffered from significantly higher overall mortality than young symbiotic beetles (Fig. 5, *P*<0.001; Table S1). By contrast, there was no difference in survival between young symbiotic and aposymbiotic individuals without exposure (controls) to the entomopathogen (*P*=0.40; Fig. S3, Table S1).

## DISCUSSION

By associating with microbial symbionts, hosts may benefit from adaptive phenotypes that can alter their interaction with environmental stressors. The grain pest beetle *Oryzaephilus surinamensis* harbours intracellular Bacteroidetes symbionts (*Candidatus* Shikimatogenerans silvanidophilus) that supplement the beetle with tyrosine precursors, thereby playing an important role in cuticle biosynthesis (Hirota et al., 2017; Engl et al., 2018; Kiefer et al., 2021). It was demonstrated that the symbiont-mediated phenotype translates to fitness benefits for the host under desiccation stress, a condition that is characteristic of the grain storage facilities that these beetles notoriously inhabit (Engl et al., 2018). However, beyond water retention, the insect cuticle has an array of additional functions, notably acting as a structural barrier against natural enemies (Hadley, 1984). Our current study shows that the nutritional symbiosis with *Shikimatogenerans* also confers enhanced mechanical defence to *O. surinamensis* against a generalist predator and an entomopathogenic fungus. Furthermore, we show that this protective effect corresponds to faster thickening and tanning of the cuticle, indicating that the symbionts enable their host to rapidly escape from the vulnerable post-eclosion phase.

Upon eclosion from the pupal case, the cuticle of insects undergoes a crucial transition from soft and white to harder and darker (Rajpurohit et al., 2021). Importantly, sclerotisation and melanisation of the cuticle are reported to coincide with a strong increase in symbiont titre within the first week post-eclosion in *O. surinamensis* (Engl et al., 2020) and in *Sitophilus oryzae* (Vigneron et al., 2014), indicating a particular need for the symbiont during cuticle formation. The impact of the symbionts on cuticle development is due to the provisioning of tyrosine precursors in both *O. surinamensis* (Kiefer et al., 2021) and *S. oryzae* (Oakeson et al., 2014; Vigneron et al., 2014), which are in high demand to produce cuticular proteins, melanin, and catecholamines used for sclerotisation (Noh et al., 2016). Concordantly, we observed a significant influence of symbiont presence and age on both melanisation and cuticle thickness during the first 7 days post-eclosion (Figs 1 and 2). Symbiotic beetles were able to rapidly develop their cuticle by increasing thickness and melanisation, while this development was slower in aposymbiotic beetles, with their cuticle never reaching the same thickness or melanisation as that of their symbiotic counterparts. Thus, symbiont-mediated cuticle biosynthesis enables symbiotic beetles to build up their cuticle more rapidly (see also Hirota et al., 2017). We then investigated how this differential rate at which the cuticle develops in symbiotic and aposymbiotic *O. surinamensis* impacts the beetles’ ability to cope with predators and fungal pathogens that need to breach the cuticle for successful attack.

The outcome of an adult beetle's encounter with a wolf spider was significantly influenced by symbiont presence or absence, with an overall higher survival probability in symbiotic beetles when compared with aposymbiotic beetles (Fig. 3). For successful predation, wolf spiders must execute a prey capture sequence that involves delivering a venom that ultimately paralyses the prey and begins the process of extra-oral digestion (Eggs et al., 2015). Thus, the low survival probability noted in aposymbiotic beetles is probably due to the reduced thickness of the cuticle, which enhances the spider's chances of successfully biting through the cuticle and injecting venom. Enhanced cuticle thickness was previously also described for symbiotic as compared with aposymbiotic beetle larvae (Engl et al., 2018), which probably explains the significantly longer spider handling times observed in symbiotic larvae, as we found no significant difference in mass between symbiotic and aposymbiotic larvae (Fig. 4). Changes in cuticular traits with progression in cuticle development were concordantly reflected in the strong age effect on adult beetle survival outcome, with survival probability increasing with a progression in age for both symbiotic and aposymbiotic beetles. However, the survival probability of symbiotic beetles increased earlier, reaching 50% around day 3, while aposymbiotic beetles achieved the same level of survival probability 2–3 days later. Similarly, Wang et al. (2018) observed that in *Pachyrhynchus sarcitis kotoensis* weevils, mature (‘hard’) weevils survived predatory attacks by *Japalura swinhonis* lizards, while young (‘soft’) weevils were easily consumed. Interestingly, symbiont-mediated provisioning of tyrosine precursors has been reported in the congeneric species *Pachrhynchus infernalis* (Anbutsu et al., 2017); thus, the escape of *Pachyrhynchus* weevils from the vulnerable post-eclosion period may also be accelerated by bacterial symbionts.

We observed a similar effect of age and symbiont presence in encounters with entomopathogenic fungi, where young beetles of both treatments suffered from significantly higher mortality than old beetles, and higher mortality rates were noted in young aposymbiotic as compared with symbiotic beetles (Fig. 5). Akin to the interaction with wolf spiders, this is indicative of underlying differences in the rate at which a cuticle quality threshold that confers protection is achieved. However, it must be noted that the adhesion of conoidal spores to the cuticle and the subsequent breach of the fungus through the cuticle are only the first steps towards successful infection (Lu and Leger, 2016). Once inside the haemocoel, the fungus must overcome the host’s immune defences (Lu and Leger, 2016). Importantly, encapsulation and melanisation constitute essential components of the insect’s immune reaction towards entomopathogenic fungi (Lu and Leger, 2016; Yassine et al., 2012). Thus, it is possible that the increased susceptibility to fungal infection of young aposymbiotic *O. surinamensis* is due to either reduced cuticle thickness and sclerotisation or an impaired encapsulation response, or a combination of the two. Interestingly, carpenter ants (*Camponotus floridanus*) harbouring tyrosine-supplementing *Blochmannia* symbionts were more susceptible to the fungal pathogen *Metarhizium brunneum* than antibiotic-treated individuals, indicating that while the symbiont is important for cuticular formation, it imposes a cost of reduced immunity upon its host (Sinotte et al., 2018). In *O. surinamensis*, recent findings also reveal a cost of symbiosis, manifested as a delay in the onset of reproduction in symbiotic beetles at low desiccation stress (Engl et al., 2020), but our results indicate that this does not have a negative impact on the beetle's immune defence against fungi.

Under natural conditions, virtually all animals must contend with natural enemies that exert strong selective pressures on individuals and populations (Oliver et al., 2014). Consequently, hosts may acquire fitness benefits from novel defensive properties conferred by their microbial partners (Flórez et al., 2015). Microbes may be involved in the direct production of bioactive compounds that deter antagonists, as exemplified by the symbionts of *Paederus* beetles that produce pederin, which deters wolf spiders (Kellner and Dettner, 1996), or the antibiotic-producing symbionts of European beewolves *Philanthus triangulum* that protect the wasp larvae from fungal infection (Kaltenpoth et al., 2005). Indirectly, symbiotic microbes may confer protection via stimulation of the host's immune system as demonstrated in mosquitos, where infection with the endosymbiont *Wolbachia* leads to increased resistance to dengue virus (Pan et al., 2012), or through resistance to colonisation by pathogens as has been found in the Oriental tea tortrix, *Homona magnanima*, and its intestinal symbionts (Takatsuka and Kunimi, 2000). By demonstrating how rapid symbiont-mediated cuticle biosynthesis post-eclosion corresponds to the early escape of symbiotic beetles from vulnerability to predation and fungal infection, this study presents a nutrition-based enhancement of structural defences as an additional way in which microbial symbionts can protect their host from natural enemies.

Grain pest beetles not only have to contend with the low ambient humidity that characterises grain storage facilities but also must evade parasitoids, pathogens and predators. The most commonly found predators that occur with *O. surinamensis* in grain storage facilities are the hemipterans *Xylocoris flavipes* and *Lyctocoris campestris*, both of which are known to attack and kill the eggs and larvae of various grain pest beetles (Donnelly and Phillips, 2001; Parajulee and Phillips, 1993). However, wild populations of *O. surinamensis* have been reported (Sharaf et al., 2008; 2013), where the beetles may encounter a wider range of small arthropod predators as well as pathogens. Furthermore, the defensive benefit of the symbiosis probably has implications for pest management because microbial pest control agents such as *B. bassiana* are now an attractive alternative to chemical pesticides against insect pests, including *O. surinamensis* (Erler and Ates, 2015). We posit that engaging in a symbiosis that supplements the precursor for tyrosine as a key compound required for cuticle biosynthesis equips *O. surinamensis* with an armour that confers desiccation resistance (Engl et al., 2018) and enhances structural defence against predation and fungal infection, providing insight into the ecological benefits that are likely to have favoured the evolution and maintenance of the symbiosis. This benefit probably extends to many other beetles in which tyrosine-provisioning microbes have been identified, including *S. oryzae*, *P. infernalis* and *E. postfasciatus* (Vigneron et al., 2014; Anbutsu et al., 2017; Anbatsu and Fukatsu, 2020; Kuriwada et al., 2010) as well as many other weevils harbouring intracellular symbionts (Zhang et al., 2017 preprint). In addition, symbionts localised in bacteriomes have been described for at least five additional beetle families (Brentidae, Bostrichidae, Nosodendridae, Throscidae and Dasytidae), and the close phylogenetic relationships of some Brentidae symbionts with *Nardonella* (Zhang et al., 2017 preprint) and of the Bostrichidae and Nosodendridae symbionts with *Shikimatogenerans* (Engl et al., 2018; Hirota et al., 2020) suggest that these symbionts may be functionally similar (Salem and Kaltenpoth, 2022). Hence, symbioses in insects can have multifaceted phenotypic impacts beyond immediate nutritional effects, and studying these more comprehensively will provide us with a better understanding of the implications of symbiotic interactions on the ecology and evolution of insects.

## Supplementary Material

Supplementary information
