# Supplementary material for: Nutritional symbionts enhance structural defence against predation and fungal infection in a grain pest beetle
Source: J Exp Biol. 2022 Jan 4;225(1):jeb243593. doi: 10.1242/jeb.243593 (PMC8778805; doi:10.1242/jeb.243593)
Supplement: Supplementary information [file jexbio-225-243593-s1.pdf]

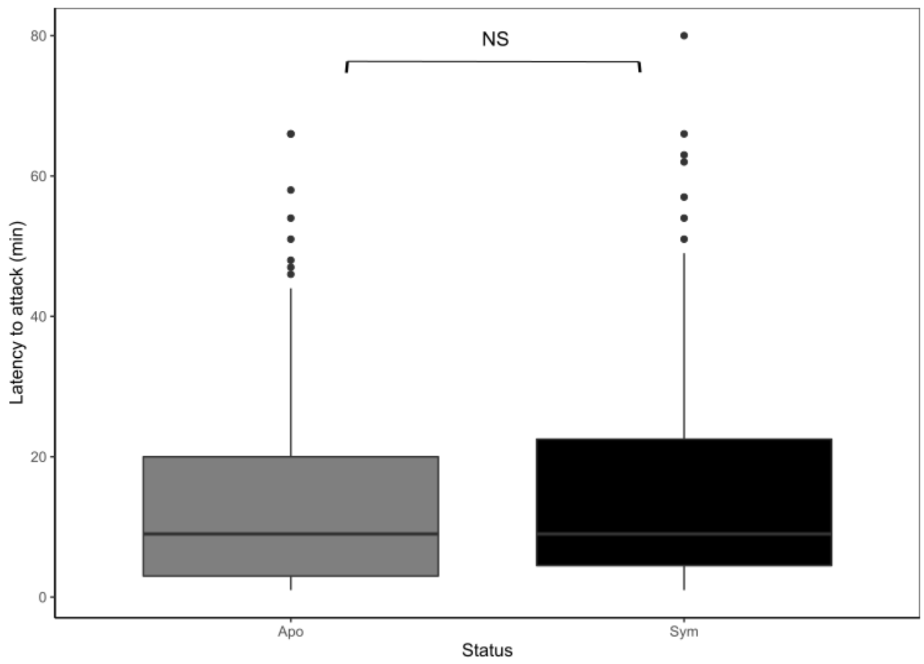

Fig. S1. The time taken in minutes (latency) for a starving spider to attack aposymbiotic (Apo, n_{apo}= 62) and symbiotic (Sym, n_{sym}= 64) beetles once introduced into the arena. Latency did not differ significantly between the treatments (GLMER: t = 0.722, p = 0.4704)

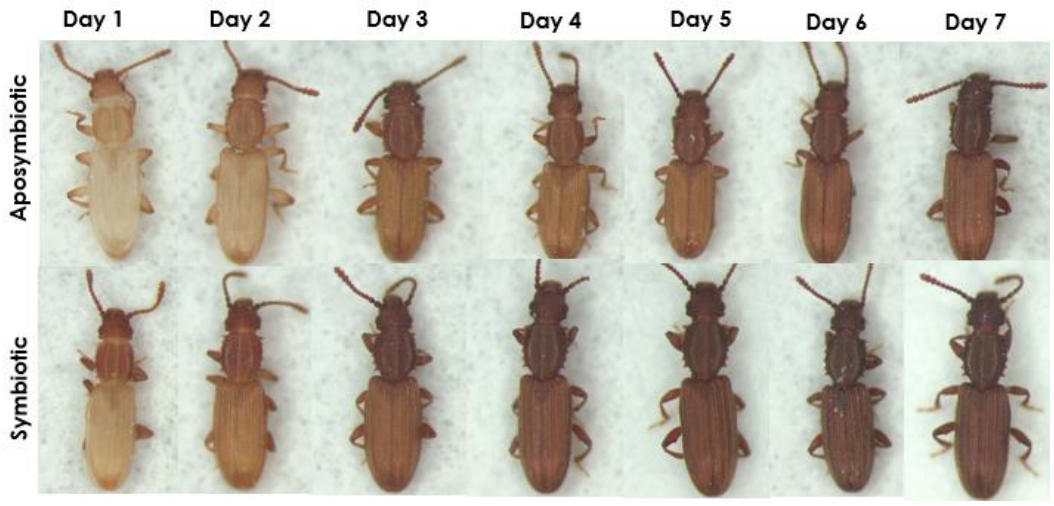

Fig. S2. Melanisation progression in symbiotic and aposymbiotic beetles from day 1 to 7 post-eclosion. Representative images of aposymbiotic (upper row) and symbiotic beetles (lower row).

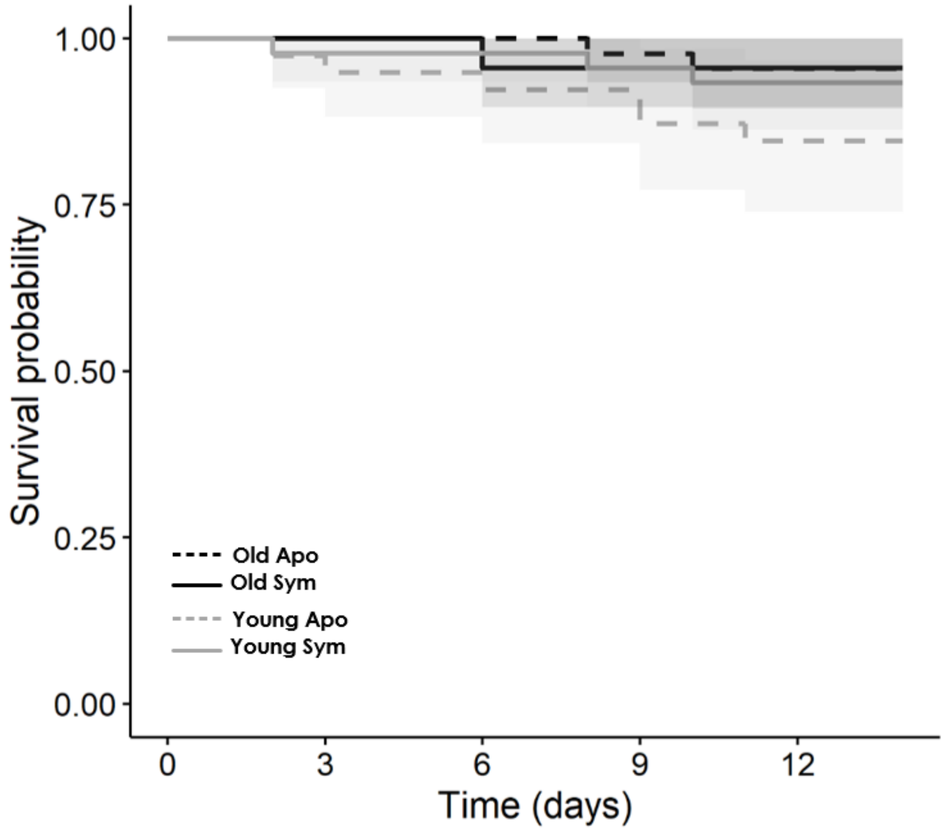

Fig. S3. Survival probability of young (<24hours post-eclosion) and old (14 days post-eclosion) symbiotic and aposymbiotic beetles without exposure to *B. bassiana* (controls). Mortality was not significantly influenced by either symbiont status or age (Cox mixed-effects model, p=0.1073; p=0.2837, respectively, n_{apo}= 45, n_{sym}= 45).

Table S1. Impact of *Oryzaephilus surinamensis'* symbiont status and age on defence against the entomopathogenic fungus *B. bassiana*. Results of pairwise multiple comparisons following COX- mixed effects models. (Sym= symbiotic, Apo= Aposymbiotic)

**Controls**		**Old Sym**	**Young Sym**
	**Old Apo**	P = 0.980	P = 0.790
	**Young Apo**	P = 0.280	P = 0.400
**Treatment**		**Old Sym**	**Young Sym**
	**Old Apo**	0.720	P < 0.001
	**Young Apo**	P < 0.001	P < 0.001
